# Building digital innovation capacity at a large academic medical center

**DOI:** 10.1038/s41746-019-0088-y

**Published:** 2019-03-07

**Authors:** Devin M. Mann, Sara Kuppin Chokshi, Rachel Lebwohl, Michael Mainiero, Catherine Dinh-Le, Katherine Driscoll, Steven Robinson, Helen Egger

**Affiliations:** 1 NYU School of Medicine, Department of Population Health, New York, NY USA; 2 NYU Langone Health, Medical Center Information Technology, New York, NY USA; 3 NYU School of Medicine, Department of Child and Adolescent Psychiatry, New York, NY USA

**Keywords:** Health care, Public health

## Abstract

Academic medical centers (AMCs) today prioritize digital innovation. In efforts to develop and disseminate the best technology for their institutions, challenges arise in organizational structure, cross-disciplinary collaboration, and creative and agile problem solving that are essential for successful implementation. To address these challenges, the Digital DesignLab was created at NYU Langone Health to provide structured processes for assessing and supporting the capacity for innovative digital development in our research and clinical community. Digital DesignLab is an enterprise level, multidisciplinary, digital development team that guides faculty and student innovators through a digital development “pipeline”, which consists of intake, discovery, bootcamp, development. It also provides a framework for digital health innovation and dissemination at the institution. This paper describes the Digital DesignLab’s creation and processes, and highlights key lessons learned to support digital health innovation at AMCs.

## Introduction

Digital innovation is a priority for many academic medical centers (AMC) looking to develop and disseminate the best of what technology has to offer their institutions.^1^ However, innovation is a tall order even in the easiest of circumstances. While AMCs are well positioned to have sustained impact on the development of new technologies and digital innovation, the complexities of an AMC, such as siloed research and operational teams and bureaucratic approval processes, pose significant challenges.^2^ Organizational structure, cross-disciplinary collaboration and creative and agile problem solving are essential for digital innovation; siloed expertise and processes stunt and slow capacity for this type of work.^2,3^

Digital health technologies are relatively new to the AMC innovation scene. The well-worn paths for fostering basic science research do not translate well to digital health.^2^ Accordingly, AMCs and digital health companies have struggled to find a way to promote digital innovation amongst their faculty.^4^ Many faculty, as well as staff and students are looking to take part in digital innovation, but are inexperienced and unclear about how to even begin.^1^

Historically AMCs are home to groundbreaking science and biotechnology development; proving grounds for new healthcare treatments and interventions.^5^ The pathway for this innovation is well developed with AMC research laboratories serving as the core vehicle for biomedical research that feed in to clear paths for commercialization. AMC technology transfer entities are expert in facilitating the knowledge transfer from basic science labs into the appropriate dissemination vehicles.^6^

At our institution, individual researchers and clinicians continue to work at the cutting edge of their fields, increasingly enabled by integrated digital tools. However, it became increasingly apparent that our institution was lacking a structured, streamlined process for enabling and supporting digital innovation in a strategic way. To address this gap, the Digital DesignLab (Designlab) was created to provide structured processes for assessing and supporting our research and clinical community’s capacity for innovative digital development. The DesignLab is an enterprise level, multidisciplinary digital development team that shepherds faculty and student innovators through a digital development “pipeline”; providing a scaffold for digital health innovation and dissemination. This case study describes the DesignLab’s creation, characteristics, and highlights key lessons learned to support digital health innovation at AMCs.

While innovation in research and clinical delivery is happening across the institution, the lack of a coordinated approach to innovation in the digital health space left our community of clinicians and researchers to fend for themselves when looking to add a digital component to their projects. They often found themselves missing the expertise, resources, or connections to get a digital tool built or tested. Moreover, the digital tools they built were typically disconnected from the enterprise IT infrastructure and therefore not designed to integrate into the enterprise healthcare delivery system. Rather than build in collaboration with operational partners from the healthcare delivery system, grant funds or other resources were often used to hire outside consultants who built one off digital tools destined to sunset with the grant that launched them.

The lack of strategy, coordination and visibility around digital health innovation meant that, often, exceptional ideas for digital tools (and sometimes even those tools that actually found their way to being built) were never positioned to reach their full potential to transform healthcare delivery. The need for a transparent, coordinated process or “pipeline” became apparent as digital tools either failed to get built or were taking unreasonably long to get built. For those that did make it through the build phase, a lack of strategic planning and support resulted in poor dissemination both within and outside of the institution. Without planning for scalability and sustainability, these tools illustrate the lost opportunity and resources that result from not having a thoughtful, coordinated approach to digital innovation.

Out of this need, the Digital DesignLab was conceived as a vehicle that would, for the first time, connect key groups from multiple disciplines in the health system to form scaffolding for a comprehensive approach to digital innovation. While the development of the DesignLab has been far from a straight line and continues to evolve, its story offers important insights into how innovation leaders in similar environments might think about solving challenges to innovation in their own institutions.

## Results

### The birth of the Digital DesignLab

The Digital DesignLab was born of the question—how might we connect and coordinate innovative research efforts involving novel digital components with our clinical and information technology operations? Our institution had a growing community of clinical researchers focused on healthcare delivery and the evolution of the learning healthcare system. These researchers and clinicians were increasingly seeking to incorporate digital tools into their innovations. For example, a successful research team received a grant to develop a digital tool focused on the child mental health space. However, the team had no expertise in digital development, and their development plan centered on finding an outside company to build their tool. We needed a process that would facilitate rather than extinguish innovation projects like this while staving off the risk of developing tools unlikely to achieve adoption even within our own institution.

In the case of this project, the team did not have plans to connect their tool to the healthcare delivery system nor were they considering the need to ensure the external vendor built a tool that was able to integrate with the enterprise IT infrastructure or was of consumer-grade standards. These gaps in digital implementation and strategy would make it difficult for clinicians and institutions to adopt, use, and disseminate their tool. Examples like this highlighted a gap in support for our innovation community; a resource that could help guide them through the digital development process and enhance their products ability to become part of real-world healthcare delivery solutions. With this in mind, our information technology leadership charged our team with developing this resource and infrastructure; supporting our innovation community and using an agile, iterative process to dynamically evolve the structures needed to meet their needs. Our answer was to bring together digitally forward research faculty and leaders in operational IT under the Digital DesignLab umbrella to unlock the potential of new digital tools to not only advance science but to improve our own clinical delivery system and serve as a model for others.

### Digital DesignLab's multidisciplinary, connected, and cross functional team

**Fig. 1:**
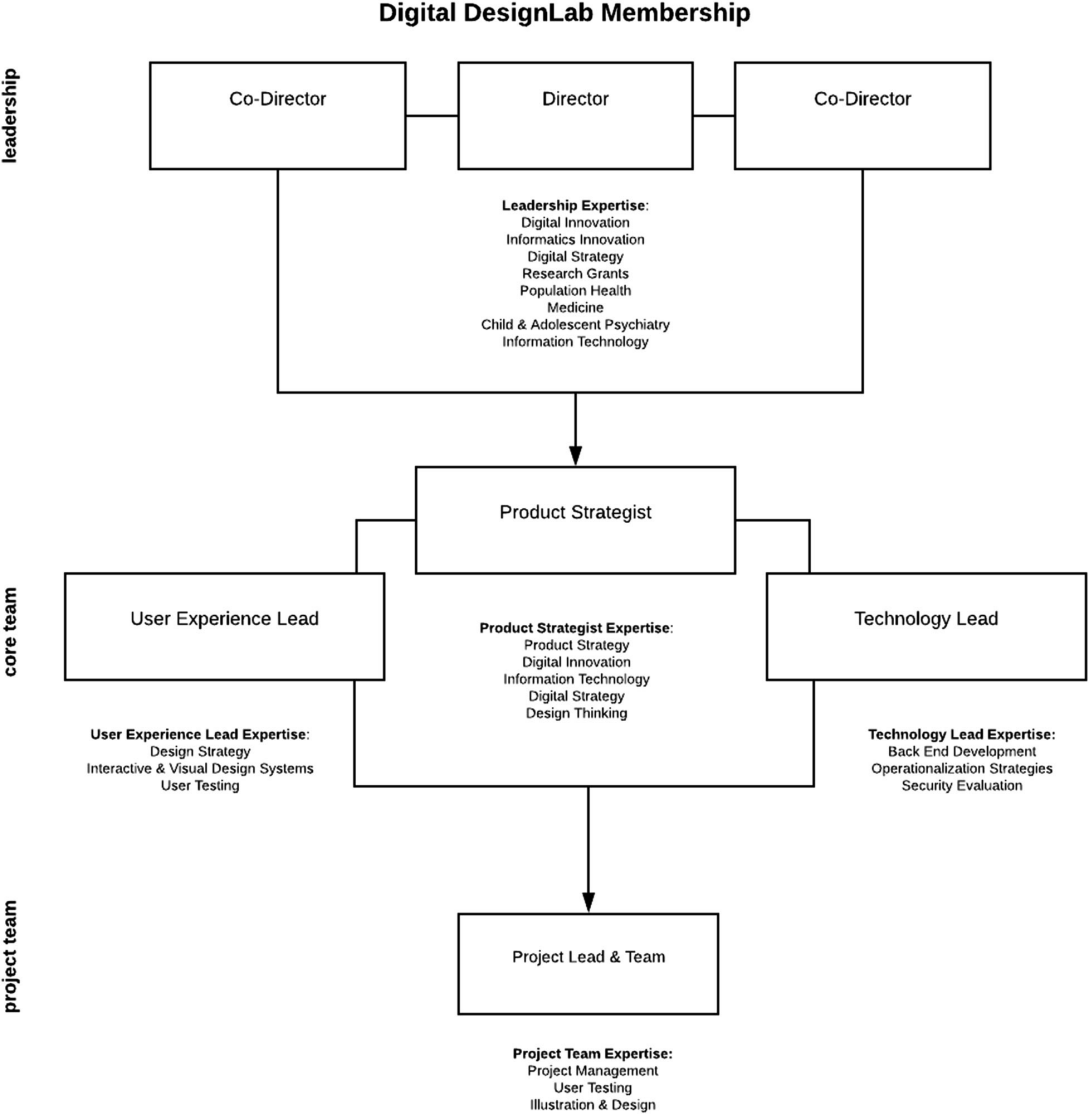
Digital DesignLab membership. The composition of the core team as well as the Digital DesignLab’s central processes are inspired by agile software development as well as lean start-up development, whose approaches are famous for their contribution to successful, user-centered digital innovation.^7,9,10^ These tools, common outside of AMCs, are used in small pockets within our institution, enabling rapid evaluation and improvement of its processes and its projects. To this end, Digital DesignLab places a priority on frequent opportunities for in-person and digital collaboration. Weekly team meetings are held and in-person attendance prioritized; similarly, daily “stand-ups” (10 min standing team meetings to discuss individual planned tasks for the day) for projects are held as appropriate.^11^ Digital collaboration resources such as Trello (web-based project management application; Atlassian, New York, NY), and WebEx Teams (collaboration application; Cisco, Milptas, CA) are used for collaborative project management, agenda setting and between meeting dialogue on project progress, “blockers,” resources and inspiration

Assembling a team that could integrate often disparate silos of expertise at AMCs was the first challenge. This required bringing together members from the academic, clinical operations, and digital solutions domains of the institution. Since the team was a digital resource, it was created to work in tight collaboration with each proposed project team. To support this organization, the team was organized into a unique structure that leveraged both the Digital DesignLab and the project team’s expertise (Fig. 1). The DesignLab is directed by leadership with deep ties and experience in clinical/implementation research, clinical operations, enterprise IT, and digital development. This group leads a core team of three roles central to digital development teams: product strategist, user experience (UX) lead, and technology lead. This core team works in close collaboration with each initiative’s project manager and content experts. The primary cost of initiating and maintaining a resource like DesignLab pertain to the fixed costs of talent that may not be there otherwise. Two of the three core team members (UX and technology lead) are full time employees sponsored by the institutional IT budget. Project management support from project teams combined with product strategy expertise from medical center IT result in cost sharing among groups collaborating with Digital DesignLab. This product/project management structure also contributes to further project teams’ commitment to their DesignLab work. This financing model reflects the institutions desire to support high level digital health innovation while fostering collaboration with research and innovation teams and expecting all members to put “skin in the game.”

The Digital DesignLab team values its cross-functional and collaborative nature as it is a key characteristic contributing to its ability to serve effectively as a resource for its projects but also speaks to its important function as a pipeline to digital development projects. Members of the team are coordinated with different hubs of digital development activity across all areas of the institution. They are also involved in regular collaboration with groups throughout the medical center participating in a wide variety of innovation-related activity, including identification of digital health startups for partnership, A/B testing of innovative uses for informatics in research, creation and piloting of cutting edge tools for medical education, and the development of digital solutions for better clinical service delivery.

### The Digital DesignLab pipeline

**Fig. 2:**
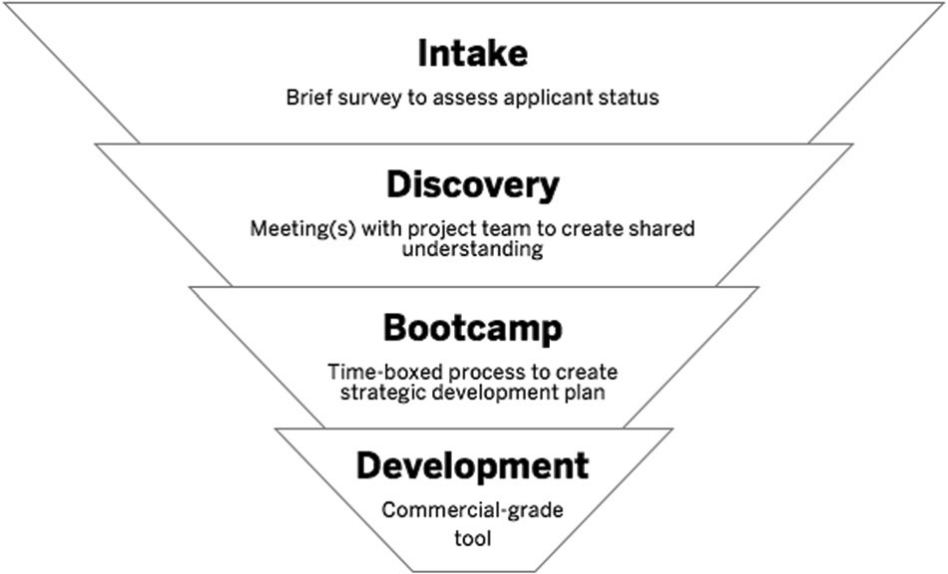
Digital DesignLab pipeline: this figure illustrates the key steps in the Digital DesignLab digital development process

At the launch of Digital DesignLab it was clear that a systematic process for identifying, evaluating, and supporting appropriate projects was critical. When research and innovation teams would reach out to team members with their ideas and requests for collaboration, no application, selection, and prioritization process existed. The lack of process hampered DesignLab’s ability to efficiently select and prioritize projects. After reviewing digital innovation processes at other AMCs, a process or “pipeline” was created to support digital health development in a systematic and transparent manner. The digital health innovation pipeline consists of four phases (Fig. 2): (1) intake; (2) discovery; (3) bootcamp; and (4) development. Every project that enters the pipeline is unique and may complete different phases than other project teams. Each phase is outlined below.

### Intake

In a large AMC, finding a path to digital development is a complex process. Many early innovation ideas lack clear definition that can exacerbate the challenge of identifying who in a large AMC is best to shepherd the idea forward. To overcome this, our team set up a simple, low burden, web-based intake form. The intake form (Table 1) has five simple questions to give the Digital DesignLab team an idea of the proposed project without posing barriers to project teams who are earlier on in the ideation phase and may be deterred by overly specific or technical questions in an online form. Once submitted, our team reaches out to the submitter to clarify and give feedback on their intake application to ensure all the information is gathered before moving to the next phase.

**Table 1:** Digital DesignLab intake questionnaire

1. What do you want to build?
2. Who is on your project team?
3. Who do you want to use your tool?
4. What is the current status of your project (what has been done so far)?
5. What kind of support do you need from Digital DesignLab?

### Discovery

After reviewing the intake information, projects deemed appropriate for further evaluation by the Digital DesignLab are moved into a discovery phase. A member of the Digital DesignLab team, selected based on skillset and the proposal, hosts an hour-long discovery session with the intake submission team to review the idea, their resources and plans for developing their digital solution, as well as other paths, if any, the team has explored to date. During the discovery meeting, key domains adapted from the lean business canvas (actionable “business” plan template based on lean principles) are discussed.^7^ Through participation in the discovery process, submitters are provided with an opportunity to begin the processes of reflecting on key elements of their idea in a user-centered way: What is the problem we want to solve with this solution (problem statement)? How will we know it is working (key metrics)? Is anyone else trying to solve this problem and how (unique value proposition)?

After the meeting, the Digital DesignLab team member summarizes the information and presents the findings to the full team at its regularly weekly meeting to discuss the proposal and create recommendations for next steps. The completion of this discovery phase is a critical juncture in the pipeline as the process encourages diverse submissions across the stages of development, from ideation, user experience design or business plans, to technical architecture and front end or back end development. A key function of the discovery is to find the right disposition for the proposal without substantial time or resource investment. Example discovery recommendations include:

1. Encouraging the team to conduct a thorough landscape review if there is a high likelihood that the marketplace (or another team at the institution) already supports a similar digital solution.
2. Connecting them to the institutional entity charged with connecting faculty with digital health startups for collaborative development.
3. Guiding them to the standardized IT optimization request process for ideas that can leverage established IT infrastructure rather than developing new tools.
4. Encouraging them to connect and collaborate with other ongoing digital development projects in similar spaces.
5. Connecting them with trusted digital development vendors that have demonstrated an ability to work with our institutional IT systems and innovation teams to produce consumer grade products.

For example, the Digital DesignLab team conducted a discovery of a mHealth sleep apnea proposal from an experienced research team. Despite their experience, our assessment revealed significant gaps in their landscape analysis and insufficient consideration of whether there was a real need for developing a new solution versus adapting an existing application. After sharing our recommendations, the research team decided to pivot their efforts into a less crowded innovation space; a “win” for the team and for NYU. By introducing efficiencies at this early, upstream stage, the Digital DesignLab amplifies benefits downstream across the entire NYU ecosystem. A subset of the intake projects that prove to be highly innovative and that align with institutional goals are considered for internal development. These projects are referred on to the Digital DesignLab “bootcamp”.

### Bootcamp

The bootcamp is a 2- to 3-month intense period of development adapted from a similar concept used at another AMC. Based on the discovery phase, the Digital DesignLab team begins this phase with the creation of a “what to expect” document that curates a rapid, digital development experience and timeline for the team (Table 2). This document draws from an inventory of digital development exercises and activities to create a custom experience to meet the needs of the proposal. Examples of activities include design workshops, landscape analyses, prototyping, usability testing, and system/data architecture and business planning research.

**Table 2:** Digital DesignLab bootcamp outline example

Timeline	3 months/12 weeks, consisting of 1–2 h/week meeting of project team with DesignLab
Product management	Kickoff (2 h)
	Feature mapping (2 h)
	Landscape analysis training (1 h)
	User testing training (1 h)
	Dissemination channel assessment (1 h)
	Business analysis/go-to-market strategy (1 h)
	Bootcamp close-out (2 h)
	Retrospective (1 h)
UX design	Design workshop (2 h)
	Secondary design workshop (2 h)
	Prototype feedback session I (1 h)
	Prototype feedback session II (1 h)
Technical	Initial technical consultation (1 h)
	Validating assumptions/scope (1 h)
	Security assessment (1 h)
	Operationalization strategy (2 h)
	Secondary technical consultation (1 h) optional

The Digital DesignLab team in collaboration with the project’s team members map out the activities required to execute each step; the teams organize a joint timeline to create a shared understanding of the goals and steps required to achieve the bootcamp goals. Activities such as feature mapping and validating assumptions help define the exact intentions of the project team’s product and if this product will accomplish their vision. A key piece of this decision-making is identifying channels of dissemination, where the DesignLab team helps the project team find the most efficient way to achieve user adoption. For example, our team conducted a bootcamp with a medical student-driven project building a behavioral intervention tool for severe itching from eczema or other dermatological conditions. While the student team had worked on it for more than a year, the intake process revealed that the product in its current iteration reflected gaps that could be addressed with some rebuilding in a more systematic and targeted fashion. The teams collaboratively conducted intensive user research and testing with low-fi prototyping to rapidly reimagine a more robust application, driving toward a minimal viable product.^8^ For more technical projects, sessions may include security assessments that can identify points of improvement in technical solutions.

The ultimate product of the bootcamp is a development “workbook,” a curated compendium of the bootcamp activities that serves multiple purposes: (1) a design document; (2) a potential “pitch” deck; (3) an artifact documenting the work of the Digital DesignLab for future bootcamps, and (4) evidence of the value of the work to its sponsors. This document is presented by the project team at a close-out session in which both the DesignLab and the project team review all progress throughout the bootcamp. At the conclusion of the bootcamp, the project has evolved substantially and often pivots to a new direction. In the case of the itching project, the student team was positioned to apply for a grant to build additional features and partner with a national foundation to explore dissemination options. Similar to the conclusion of the discovery, the team once again makes a recommendation for next steps that may include partnering with internal or external teams working on similar products. A small proportion of the bootcamp products will be strategically aligned with institutional goals and represent a solution that would benefit from an in-house development trajectory. In those cases, the project migrates into a development phase under the DesignLab umbrella. At the end of each bootcamp, the DesignLab team meets for a retrospective to review the bootcamp process and discuss what worked, what did not work, and steps to improve the process in the next bootcamp.

### Development

Internal development of digital solutions is a resource intensive investment. Each internal development project needs to be carefully reviewed for its potential cost and value to the institution. Once in development, the project moves into a lean startup style phase, working in sprints supervised by DesignLab members comfortable in agile development structures. Typically, the DesignLab team supervises the product strategy, user experience and technical architecture while any software development or coding (if relevant) is outsourced to more cost-effective entities. While timelines can vary, six to nine months is the standard expected timeline for the development of a working prototype or beta of the tool. Throughout the development period, the team works closely with the research/innovation team as well as the institutional technology development teams to build the digital solution's dissemination plan, including recommended channels, to ensure that the product is optimized for success and impact. For example, our team is currently preparing the launch of an application that helps parents understand and manage their children’s picky eating behaviors, while providing researchers new insights into this difficult to treat behavior. In its bootcamp phase the project defined its key features, honed its technical architecture to support parents’ and researchers’ needs, and visioned a go-to-market strategy. During the build phase, development sprints were conducted in collaboration with an offshore software development team to rapidly assemble a first draft of working software (a.k.a. minimally viable product). When to Wonder: Picky Eating is preparing for a national launch to parents, clinicians and researchers invested in treating this condition (currently available on the App Store or Google Play http://l.ead.me/bb2UvH).

### Project selection and evaluation

Digital DesignLab works in the spirit of innovation, therefore individuals who bring their projects to DesignLab are asked for feedback about process through a common Agile software development evaluation process called a “retro” (retrospective) in which teams review and discuss process systematically at key intervals to identify areas for process improvement going forward.

Weekly meetings contain dedicated time to discuss among the DesignLab team the health of projects. Additionally, the agile approach to project completion by design has built in tools for self-reflection for the project teams, offering opportunities for iteration and pivots. DesignLab has a “What to expect from bootcamp” document that outlines expectations for project teams and discussion of possible termination of support for those who do not satisfy criteria (e.g., devoting sufficient time and commitment for project completion).

Institutionally, Digital DesignLab collects process metrics, including project inquiries, completed intakes, projects diverted to other more appropriate resources, project bootcamps completed, and products launched. In the future, we anticipate collecting data on financial contribution of DesignLab via grant dollars captured for relevant projects that have DesignLab as a resource, as well as reach.

### Products from year 1

In year 1, Digital DesignLab received 67 intake forms, resulting in 36 discovery meetings, 6 bootcamps, and is currently about to graduate its first development project; these represent 7 schools and institutes (e.g., business, engineering, education, medicine) and over 20 different departments. Digital DesignLab has been key in bringing together various operational and research groups doing work in digital innovation to coordinate efforts and is part of a newly launched institution-wide umbrella group aimed at addressing a wide variety of aspects of digital innovation from innovations in medical education to scouting new technologies, to research related to implementation of innovative digital tools. At our institution’s annual internal health technology symposium, Digital DesignLab hosted an event in collaboration with other research and IT groups, in which 52 project teams reached out to DesignLab for advice on next steps for their digital innovation projects or ideas; 40 teams completed consultations at the event.

## Discussion

The thirst for innovation and the desire for guidance and support in the process within our AMC is exemplified by the response to Digital DesignLab thus far; however, the first year of Digital DesignLab has had its challenges and missteps. For example, it quickly became evident that the development of a systematic process for triaging and prioritizing projects is key. Additionally, as the Digital DesignLab team itself became accustomed to working in an agile way, our experience with project partners accustomed to the slower cadence of research taught us the importance of selecting project partners capable of devoting the time and depth of energy required for bootcamp. To this end, we now provide ample information about expectations from the outset (as reflected in the “What to expect from bootcamp” document). Plans for funding and sustainability are also at the forefront of decisions about a projects appropriateness for bootcamp or development with Digital DesignLab. Noticing potential project partners typically came to DesignLab with little knowledge of the landscape of existing tools or technologies similar to their idea, moving forward we plan to increase our capacity to incorporate market scans into the project selection process.

Due to the fact that its leadership comes from the key areas of enterprise IT, clinical operations and research, Digital DesignLab was able to successfully develop a process to assess and select projects based on a multifaceted assessment of their alignment with the DesignLab mission and institutional priorities. Projects are selected based on the likelihood that resulting innovations would be useful for the institution which creates efficiencies explicitly. This structure has been institutionally transformative, breaking down long standing silos that were constraining digital health innovation in our AMC which has been key to the success of Digital Designlab thus far. This aligned, coordinated approach increases our institution’s capacity to prioritize support of projects that are likely to achieve integration at the enterprise level. In this vein, Digital Designlab leadership is spearheading efforts to initiate use of an EHR-integrated platform that would allow clinicians to prescribe digital applications to patients; leveraging this platform would decrease the time and resources necessary for EHR-integration increasing odds that these products achieve adoption at our institution.

The Digital DesignLab team, with members from a diverse array of relevant backgrounds and leadership with expertise and interests in technology both from a research as well as an operational perspective, provides both key knowledge and institutional situationality needed to dissolve key barriers to agile innovation. While innovation centers are becoming increasingly common in AMCs, the DesignLab is unique in its cross-departmental and research-operations collaboration (as reflected in its leadership), and its commitment to prioritizing good, user-centered design in support of highly useful, useable tools that are well-placed for integration into real world clinical settings.

The return on investment of the Digital DesignLab developed throughout its first year and continues to reveal itself. Initially, Digital DesignLab’s contribution has been in the form of intellectual capital (technical and process support) necessary to increasing capacity for innovation. This kind of work also contributes the institution’s reputation as cutting edge and forward thinking. Over time, Digital DesignLab has served a crucial role creating a cohesive digital innovation community at the medical center, bringing together likeminded clinicians and researchers for increased collaboration and synergy. The DesignLab is now an important resource for research grants, serving as an indicator to funders that the institution has the facilities and resources needed to support truly innovative projects. The DesignLab has produced innovative technological capability that translated into the core of a recent peer-reviewed grant proposal; an EHR-integrated machine learning algorithm for personalizing decision support that began as a DesignLab project is currently the basis of a grant proposal under review. DesignLab has also provided advice and content for researchers who are preparing grant proposals to initiate innovative digital development as in the case of a researcher looking to build a platform to study the use of behavioral economic nudges to influence online food shopping choices for recipients of food assistance program benefits.

We plan to incorporate formal educational opportunities for clinicians, researchers and students in the coming year; currently, partnerships with project teams whether in the context of bootcamps or grant development involve a component of education, leaving project teams with greater understanding and capacity needed to execute digital innovation projects. While it remains to be seen whether Digital DesignLab will be a self-sustaining entity or become an operating cost, as illustrated above, while not always explicit, the value created by Digital DesignLab in the form of raising the profile and capacity for innovation for the institution make it an increasingly worthwhile endeavor.

### We built it and they came

The Digital DesignLab is a promising approach to fostering digital health innovation at a large AMC, making innovation accessible by allowing more people to participate in creating healthcare solutions and unlocking the full innovation potential of the enterprise. The pipeline demystifies the journey to creating innovative digital health solutions and allows faculty, students, and staff from all sectors of the healthcare enterprise to engage and collaborate in innovation. The visibility provided through the pipeline helps not only potential innovators but also provides an opportunity to the institution itself to systematically coordinate and prioritize digital development and promote the rapid development of new digital health tools with the potential to change healthcare.

## Methods

Presented as a case study, we describe the Digital DesignLab’s creation, characteristics, process, and key outcomes in its first year. The discussion highlights key lessons learned from this case study to support digital health innovation at AMCs.

### Reporting summary

Further information on experimental design is available in the Nature Research Reporting Summary linked to this article.

## Supplementary information

Reporting Summary

## Data Availability

No data were generated or analyzed during this case study.

## Supplementary information

**Supplementary information** accompanies the paper on the *npj Digital Medicine* website (10.1038/s41746-019-0088-y).
